# Present Food Shopping Habits in the Spanish Adult Population: A Cross-Sectional Study

**DOI:** 10.3390/nu9050508

**Published:** 2017-05-18

**Authors:** María Achón, María Serrano, Ángela García-González, Elena Alonso-Aperte, Gregorio Varela-Moreiras

**Affiliations:** 1 Department of Pharmaceutical and Health Sciences, Facultad de Farmacia, Universidad CEU San Pablo, Alcorcón, 28925 Madrid, Spain; achontu@ceu.es (M.A.); maria.serranoiglesias@ceu.es (M.S.); angargon@ceu.es (Á.G.-G.); eaperte@ceu.es (E.A.-A.); 2 Spanish Nutrition Foundation (FEN), 28024 Madrid, Spain

**Keywords:** Spain, food shopping, grocery shopping, shopping responsibilities, fresh produce shopping, food store types, shopping patterns

## Abstract

Information on grocery shopping patterns is one key to understanding dietary changes in recent years in Spain. This report presents an overview of Spanish food shopping patterns in the adult population. A cross-sectional, nationally representative telephone survey was conducted in Spain. Individuals were asked about food shopping responsibility roles, types of visited food stores, time spent, additional behaviors while shopping, the influence of marketing/advertising and, in particular, fresh produce shopping profile. Binary logistic regression models were developed. The final random sample included 2026 respondents aged ≥18 years, of which 1223 were women and 803 were men. Women reported being in charge of most of the food shopping activities. Looking for best prices, more than looking for healthy or sustainable foods, seemed to be a general behavior. Supermarkets were the preferred retail spaces for food price consideration, convenience, variety and availability. Fresh produce shopping was associated with traditional markets and neighborhood stores in terms of reliance and personalized service. It is essential to highlight the importance of the role played by women. They are the main supporters concerned in preserving adequate dietary habits. Economic factors, more than health or food sustainability, are commonly considered by the population. Traditional markets may play an important role in preserving some healthy dietary habits of the Mediterranean food culture in Spain.

## 1. Introduction

Understanding eating patterns, including the context of food shopping and consumption, can improve the design of programs and policies targeted at improving overall nutrition and, more generally, at informing consumer education, food assistance programs, and product development marketing. 

Furthermore, even though current societies are the most developed and informed, there is still uncertainty about what specific dietary recommendations should be followed. Official public health recommendations coexist with the overwhelming effect of advertising, thus leading to contradictory messages for the average consumer and, paradoxically, making food shopping more difficult today than it was some decades ago [1,2]. 

Some important social factors may have influenced the changes in actual food habits in Spain. One is the ageing of the population, as life expectancy has steadily increased in Spain; at the same time, birth rates have dramatically decreased during the last fifteen years [3]. The implications of such ageing are definitely concerning, since the elderly is one of the population groups at highest risk of malnutrition; there are multiple factors determining different dietary patterns and finally nutritional status [4]. Disabilities become more common as age increases; autonomy is reduced and routine activities such as food shopping, carrying bags, preparing food, or even the fact of eating itself, are limited [5,6]. The consequences, together with problems of chewing, constipation, high prevalence of degenerative diseases, and consumption of drugs, are monotonous and unbalanced diets, with low intakes of nutrients such as certain vitamins and minerals [7,8,9,10]. Actually, in Spain, about 4% of the population ≥65 years are malnourished and 22–25% are at risk to suffer it. The risk is higher in women than in men [11]. Secondly, the incorporation of women into the workplace has affected general housework activities, specifically those related to feeding the family, a primary component of housework that has traditionally been the responsibility of women in Spain [1] as well as throughout the Mediterranean region and other developed countries [2]. A generalized incorporation of females into the active workforce involves a lower purchase of all food groups, probably due to eating out more frequently [12]; this transition has been recently considered as one of the reasons leading to partially moving away from the traditional Mediterranean diet [13]. In Spain, currently, according to The National Statistics Institute (*Instituto Nacional de Estadística,* INE), women spend 105 min a day performing cooking activities (preparing and serving meals; actually feeding members of the family; cleaning up after meals, etc.), while men spend an average of only 55 min on these tasks [14]. So even though there seems to be a trend showing a reduction in the time spent by women and an increase in the time spent by men in the last decade [15], it is important to see if women continue to conduct most of the food shopping. Third, the recent economic crisis has exacerbated a shift in food shopping habits, increasing purchases in supermarkets and supercenters (considered as all those food stores more than 400 m^2^), compared to other types of stores, particularly in terms of “fresh” produce. Since the crisis began, supermarkets have increased seven share points versus fresh produce canal specialists (traditional markets and neighbourhood food stores), rising from 51% in 2008 to 58% in 2014 [16].

Most of the studies on food habits/nutrition in Spain have basically focused on what and how much food we eat. There are many epidemiological nutritional studies that, by using different methodologies, report the diet composition, energy and nutrient intake, food portions, food frequency, and also anthropometry and biochemical indicators or physical activity and, therefore, can report the consequent energy balance [13,17,18]. Other studies are mostly economic and commercial studies that focus on how much money the Spanish population spends on food and related activities [19]. However, neither the nutritional nor the economic studies describe the Spanish food shopping model in sociological terms, even after Spanish and, in general, Mediterranean dietary guidelines have recently included, as their basis, key recommendations on social aspects related to food intake and other healthy lifestyles. These new guidelines are targeted to increase people’s concern for food sustainability, healthy and traditional cooking methods and conviviality, which were not included in previous editions of the guidelines [20,21]. 

Food pattern transformations in Spain have been proposed to be supported by lifestyle changes, leading to inadequate dietary patterns [1]. Knowing what specific social factors are changing and how and why they are changing are large challenges. The present research aims to highlight updated information about present food shopping habits in a representative sample in Spain, and their conditioning factors in different age groups, with a special focus on shopping for “fresh” products. The reason for the latter focus is that fresh produce intake is an important part of Mediterranean dietary habits, and these products are usually considered healthier when compared to processed/ultra-processed foods. 

## 2. Materials and Methods 

### 2.1. Sample and Study Design 

The study was designed as a cross-sectional survey of a nationally representative sample of adults (≥18 years old) living in Spain. A questionnaire of 55 items was specifically developed for this study: 16 items focused on sociodemographic aspects, 8 focused on food eating patterns, 18 focused on shopping habits, 9 focused on cooking habits and skills, and 4 focused on nutrition knowledge and perception. The survey was randomly administered by telephone by professionally trained surveyors and completed by individuals in private dwellings, in all territories of Spain, except for the Canary Islands and the Autonomous Cities of Ceuta and Melilla in North Africa. A pilot test was undertaken to guarantee the optimum design and adequacy of the questionnaire prior to its definite validation. Among the 119 telephone calls made, there were seven respondents who completed the pre-test. The surveys had an average duration of 22 min. Fieldwork was undertaken in May 2015, and a total of 31,552 phone calls were made.

Once the respondent was enrolled in the survey, non-response rate to questions was very small (1.5% as an average, and a maximum of 29%) thanks to the experience of the surveyors, the type of questions, the methodology used (phone call), and the automation of the process (using Computer Assisted Telephone Interview (CATI) software); all of which allowed for a better collection of data. In order to complete the questionnaire, volunteers were contacted several times, at different times of the day, until the questionnaire was completed. Those questionnaires with an elevated number of blank questions were rejected for analysis. 

Sampling was stratified, setting a minimum for the strata where the universe was smaller, with a random selection of individuals to be surveyed. The final distribution presented structural differences with respect to the actual population because the affixation of the sample by strata was carried out in a non-proportional manner, in order to ensure the representativeness of certain strata. Therefore, it was necessary to apply a weighting factor to save the proportionality of each of the strata of the sample with respect to the actual population under study. This weighting was carried out according to the following variables: sex, age and Nielsen area. 

Verbal informed consent was obtained from all subjects; data were collected anonymously and recorded according to the Spanish Organic Law of Personal Data Protection (LOPD) 15/1999.

### 2.2. Data Classification and Analysis 

Respondents’ sex and age, habitat size and geographical area of residence were requested in the survey. Based on census data published by The National Statistics Institute (INE) since 2012, ages were categorized into the following groups: 18–30, 31–49, 50–64, 65–75 and >75 years. Habitat size was divided into the following strata: less than 2000 inhabitants, 2000–10,000, 10,000–100,000, 100,000–500,000 and more than 500,000 inhabitants. Geographical area stratification considered the eight so-called Nielsen areas, according to the eight geographical areas previously outlined by annual Spanish National Food Consumption Surveys [22]: Northeast and Balearic Islands; East Coast; South; Centre of Spain; Northwest; North; Barcelona (Metropolitan Area) and Madrid (Metropolitan Area).

### 2.3. Statistical Analyses 

All variables are categories, and the results are described as frequencies and percentages. To show differences in behaviours between gender and age groups, a binary logistic regression model was applied, with “female” and “over 75” as residual categories, according to previous literature [1]. For those results displayed as distributions, differences between groups or variable distribution were evaluated by using a chi-squared test (z-test for multiple comparisons). 

Due to low non-response rate, analysis was made by means of efficacy, not taking into account missing values. For those questions with a higher no-response rate, differences in main characteristics (gender, age) were analyzed between respondents and non-respondents. No statistical difference was found between both groups, for any of the presented variables. 

For all statistical analysis, differences were considered significant at *p* < 0.05. Statistical analyses were performed using SPSS v.20.0 (IBM Corp., Armonk, NY, USA).

## 3. Results

A total of 31,552 phone calls were made, and the final participation rate was 6.42%. In total, 54.7% of the surveys were completed on the first attempt. The final sample included 2026 respondents aged ≥18 years, of whom 1223 were women and 803 men. By age group, 320 respondents were between 18 and 30 years old, 801 were between 31 and 49 years old, 460 were between 50 and 64 years old, 218 were between 65 and 75 years old, and 227 were older than 75 years old.

The sampling error was ±2.18% for global data, calculated for a confidence interval of 95.5% (CI 95.5%) and *p* = *q* = 0.5. 

To determine the main person responsible for carrying out most of the food shopping activities, we performed a binary logistic regression model in which the dependent variable took the value “1” if more than 50% of food shopping was carried out by that person and “0” if not. The independent variables were sex and age (Table 1). Women reported having the main responsibility for doing most of the food shopping more frequently than men. When evaluating the age effect, we observed that people aged 31 to 64 years old more frequently reported being in charge of most of the food shopping; young adults (18 to 30 years old) were not the main persons responsible for food purchasing.

In addition, women spent more time than men doing grocery shopping (Table 2). In this case, the dependent variable took the value “1” if food shopping took 60 min or more and “0” if it took less than 60 min. The considered independent variables were again sex and age. In this case, the younger the person, the higher the probability of spending more than 60 min on food shopping.

We also sought to gain an understanding of household food shopping habits in terms of where people decide to shop and why. The differences were evaluated among the main three types of food stores in Spain [16], and foods were classified into “fresh” and “non-perishable” (Table 3 and Table 4). Supermarkets and supercentres were the preferred place for purchasing both fresh and non-perishable foods (48% of participants bought fresh food and 90% purchased non-perishable food at supermarkets). In this item, no differences were observed between males and females, as shown in Table 3. Supermarkets were also the preferred store in all age groups, especially in the youngest group, where the lowest percentage of preference for the traditional market was found (*p* < 0.05 vs. neighbourhood stores and versus supermarkets/supercentres). Volunteers over 50 years do not show different preferences for traditional markets or neighbourhood stores when buying fresh food products, but they prefer neighbourhood stores when buying non-perishable food items (Table 3). For each type of store, no statistical differences were found for gender, or between the different age groups, compared to the reference age group (those >75 years).

For fresh products, supermarkets and supercentres were the most frequented stores in terms of the following criteria (Table 4): proximity to home, price, convenience/home delivery, and variety. Traditional markets and neighbourhood stores are more frequently visited when the criteria of food quality, origin, reliance or personalized service are considered. The food quality is, actually, the most considered reason regardless of the type of food store. For non-perishable foods, traditional market is also the main option considering food quality. Reasons such as proximity to home, price and availability do not affect customers in their selection of the type of store for non-perishable foods. No differences have been found among the reasons for buying in one or another type of store, according to gender or age groups.

Food shopping does not only involve adding products to the “basket”. Many other activities are also associated with shopping itself. Therefore, another model of binary logistic regression was developed, with the dependent variables taking the value “1” if the indicated activities were generally carried out while shopping, and “0” if not. The independent variables were sex and age (Table 5). In comparison to men, women more frequently check the “best before” date labels, compare prices, look for food price discounts and coupons while purchasing, and buy more environmentally friendly foods. However, there are no significant differences by gender for activities such as reading nutrition fact labels or looking for healthier foods. Regarding age, in comparison to people over 75 years old, all age groups are more likely to compare prices and look for food price discounts and coupons. The 18–64 years old groups usually read nutrition fact labels more often, whereas looking for healthier food seems to be less important for participants under 30 years old. Unexpectedly, it is important to highlight that people over 75 looked for environmentally friendly foods more often than young adults (*p* = 0.018), and that the group that showed a tendency (*p* = 0.053, not significant) to buy environmentally friendly food was the group aged between 65 and 75 years old.

Fresh produce is considered essential to the Spanish shopping basket, according to traditional Mediterranean dietary habits [16,19]. When studying the frequency of fresh produce shopping by binary logistic regression (Table 6), the dependent variable took the value “1” if the fresh produce shopping was performed on a daily basis and “0” if it was performed weekly or bi-weekly. The independent variables were sex and age.

There are no significant differences by gender, and when considering age, adults 18–49 years old generally buy fresh produce weekly or bi-weekly, rather than daily, compared with the oldest population group. No significant differences were observed for the other age groups.

When comparing the frequency of fresh produce shopping by habitat size (Table 7), no differences were observed. In this regard, it is remarkable that there is a high percentage of the population (83.3–92.3%) who do fresh produce shopping weekly or bi-weekly.

The preferred types of food stores selected when doing fresh produce shopping, according to Nielsen zones, were also evaluated (Table 8). Once again, the supermarket is the preferred retail space for shopping in all zones, particularly in northwest Spain. The metropolitan areas of Barcelona and Madrid have a high average population (30–32%) who purchase fresh produce in traditional food markets. With regard to the preferred food stores by gender or age groups, no significant differences are found.

To study the influence of advertising on grocery shopping, the model of binary logistic regression included the dependent variables that took the value “1” if the population was generally influenced by both traditional and Internet types of advertising, and “0” if not. The independent variables were sex and age (Table 9). No differences were observed by gender, in terms of influence of Internet or traditional advertising on food shopping. There was a significant influence of Internet advertising on food shopping mainly in young and adult people (aged 18–64 years old, in comparison to people over 75). Differences concerning the influence of traditional advertising on food shopping are significant for the young and young-adult populations, 18–30 and 31–49 years old, but not for people aged 50 years or more.

## 4. Discussion

Our results clearly show that in Spain today women do most of the food shopping. This pattern maintains the Spanish tradition, according to which women are the main players involved in food-related activities. In addition to Spain, other highly developed countries also show this trend: in the USA, 73% of women (aged 25–64) reported that they usually did the grocery shopping for their household [23], whereas only 32% of men reported having this responsibility. Therefore, despite the increasing incorporation of men into food-related activities [24], it seems that women have stayed mostly in the same situation as that twenty years ago, when they still reported doing at least twice as much work in the home [25].

When evaluating the age effect, we observed that people aged 31–64 years old are also in charge of most of the food shopping, while young adults (18–30 years old) and people aged 65 or over are not. Once again, the same pattern occurs in Americans: those aged 18–24 were more likely to report that they were not the usual grocery shopper in the household [23]. It is interesting to note that in Spain, the average age when leaving the parental home is 28.9 years old [26,27]; according to statistical data [27], 58.2% of youths between 25 and 29 years still live with their families, a percentage that is still high (26.8%) in young adults aged 30–34 years. Therefore, it is possible that the mother is the one in charge of most of the purchasing. It could also be hypothesized that when these young adults become “independent” (31–49 years old), they have to take responsibility for their own purchases, though further studies are needed to confirm this hypothesis.

For all age groups under 75 years old, it was reported that food shopping took at least 60 min. This result is consistent with the Spanish Survey of Time Use [14], which shows that the average time spent in household purchases was one hour, no matter if the purchase was made on weekdays or during weekends. In the USA, the average time spent in grocery shopping is 44 min, and women even spend at least five more minutes (46.2 min) compared to men (41.0 min) [23]. 

In recent years, and exacerbated by the economic downturn, a major transformation in the scene of food distribution and retail facilities in Spain has occurred. The shifts in citizens’ consumption habits and the impact of the economic crisis have drawn a new reality, where supermarkets are strengthened, and in fact, are the only channel that continues to grow versus the traditional trade. Supermarkets are leaders in the sector, accounting for 72% of sales and increasing. Specifically, at the end of 2013, in Spain, supercentres that maintained their numbers were virtually unaffected (444 stores); large supermarkets increased their market by adding 100 new units, and smaller ones also grew in number, basically through the franchise formula. On the other hand, other channels decreased in the number of establishments. The Spanish population at present enjoys shopping in food commercial areas with a good price/quality ratio, where a great assortment of products can be found, which thus leads to a positive shopping experience [19]. 

Our results also show that supermarkets and supercentres are mainly chosen by the Spanish population to do the grocery shopping for both fresh and non-perishable foods. This result seems to agree with the new macro trends affecting today’s food shopping culture. The proximity to home, the price, and the convenience as well as variety and availability are important criteria in choosing these retail spaces, similar to other studies where food shopping patterns have been evaluated [28].

There is an interesting effect related to fresh produce shopping: food quality, personalized service and reliance are important factors in choosing traditional markets or neighbourhood stores in this case. There seems to be a cultural tradition that links the purchase of fresh produce to traditional markets. Actually, this is one of the reasons why Spanish Dietary Guidelines are represented by the so-called “Traditional Healthy Market”, a familiar image (easy to remember and implement) for most of the population [29]. Our results are consistent with those also previously reported in other countries from the Mediterranean context such as Portugal and France, where some fresh foods, such as vegetables, fish and meat, are also purchased more at the traditional retailers than in supermarkets; however, the purchase of some other perishable goods (fruits) in supercentres is also becoming considerable in this Mediterranean environment [30,31,32]. 

It is well known that access to stores closer to the shopper may promote more frequent shopping and the consumption of fresh produce and, more specifically, fresh fruits and vegetables, key components of a healthy diet widely associated with reduced chronic diseases and optimal weight management [33,34,35]. Our results in this sense are particularly important concerning metropolitan areas, where an important average percentage of the population (32%) purchases fresh produce in traditional food markets. These types of traditional and proximity stores could therefore play an important role in the preservation of our Mediterranean dietary habits, which are declining in Spain, with the increased intake of high-lipid and high-protein food sources of energy (meats and derivatives, baked goods and pastry) and insufficient intake of cereals and derivatives for adequate nutrient density (i.e., carbohydrates and dietary fibre intake) [13].

While shopping frequency has changed over the years in Spain, our data reveal that most of the population makes purchases of fresh produce weekly or bi-weekly. No differences were found for gender or habitat size, but adults over 50 tend to buy fresh food products more frequently on a daily basis than younger ones. In this sense, our data are consistent with those previously published that reported that more than 58% of the population does the fresh produce shopping once or twice a week [24]. 

Women are more likely to be more conscious of the act of buying food. They report spending more time at the grocery store than men, and they also report that they are concerned about doing activities such as checking the “best before” date labels, comparing prices, looking for food discounts and coupons, and buying more environmentally friendly foods compared to men. 

To compare prices and to look for discounts are common practices in all age groups. The economic crisis that Spain has been undergoing since 2008 may be clearly related to the latter. Actively seeking promotions and discounts is actually one of the behaviours that has become more common in Spain in recent years. In fact, 45% of the population report seeking discounts, and 31% admit having visited a new store especially for discounts. There is a hyper-sensibility to price: 67% of the population is fully aware of the cart price and the ups and downs of prices [16]. In our sample, there is also a reasonable interest in nutrition fact labels, shared by the population in general; however, young adults aged 18–30 are not likely to look for healthier and/or environmentally friendly foods. This might be somehow shocking, considering that this result could mean they are not concerned enough about their health or even about the planet’s sustainability. The results about sustainable shopping are aligned with other surveys made by our research team on the knowledge of and attitudes toward food sustainability of young people [36]. Another message that could be inferred from our sample is that although young adults read nutrition fact labels, this does not really lead them to make healthier food choices, as the recent data on food consumption and dietary patterns show in Spain [19]. 

Currently, shopping and eating behaviours are inexorably linked within the context of modern food culture, with social media influencing consumer food behaviour [19]. There is an Internet boom, but traditional advertising continues to be the preferred mode. Mass media, such as TV, radio, daily newspapers and magazines, as well as posters in public spaces, calls at outlets (merchandising), and commercial mail received at home are included in traditional advertising, versus new online methodologies. In our study, the most important effect of advertising has been observed in the young and young-adult population (aged 18–49) that is likely to be influenced not only by traditional but also by online advertising and marketing. From our research, it is not possible to determine if this influence may have an impact on the lack of awareness in making healthy/sustainable food choices, but other research studies have proved that there is a relationship between advertising and bad eating habits, especially in children [37]. Our results seem to follow the present trends for the next decade; the proliferation of online shopping seems to be one of the new and rapid consumer trends. Marketplaces, where consumers can find products from anywhere in the world, proliferate, and there is a transition between virtual and real purchases that is becoming increasingly popular [38]. In our sample, the reported influence of online and traditional advertising can be considered as a typical characteristic of developed and consumer societies, also sensitive to new trends affecting food culture today. 

Our results show that food shopping patterns remain stable and similar to those of developed countries. However, studies on eating habits in our country show an important change in the traditional Mediterranean diet pattern, while the prevalence of obesity and other non-communicable diseases keep increasing [39].

Strengths and limitations of the present study. The present study shows updated results from a nationally representative sample, within a wide age range, and includes the main determinants of food shopping habits in Spain. The main limitation is that the results are expressed on a cross-sectional basis and do not reflect a possible evolution during a specific period of time. Other limitations include the low participation rate, and the assumed representativeness of the different age groups. The validation of the survey is only based on reproducibility and face validity.

## 5. Conclusions

When analysing grocery shopping habits in Spain, it is essential to highlight the importance of the role played by women, who remain responsible for most of the food shopping tasks. They are the ones who spend more time on this activity, and are the most concerned in seeking appropriate prices, checking “best before” date labels, and also buying sustainable foods. They are thus the main supporters potentially involved in preserving adequate dietary habits.

Supermarkets are the preferred retail spaces for grocery shopping for both fresh produce and non-perishable foods, regardless of sex, age, geographical area or habitat size. Consumers choose these types of stores because they are considered to offer quality, comfort, price, variety, and availability. Fresh produce is considered an important component of grocery shopping. Its purchase is related to traditional markets and neighbourhood stores in terms of food quality, reliance and personalized service, otherwise considered key points by shoppers to select these proximity stores. There is a major effort by young adults to try to buy fresh produce frequently, a strength that can help maintain some healthy dietary habits of the Mediterranean food culture of our country.

Activities such as price comparison or looking for discounts while shopping are shared by the majority of the Spanish population. In fact, these activities seem to be a general behaviour, much more common than activities such as looking for healthy and/or sustainable food, which are not likely to be usually considered by young adults. A large part of the population also report being influenced by advertising, both traditional and online, at the time of purchase. This influence reflects that our representative adult sample is a society that seems to fit the new proposed consumer trends for the next decade. 

## Figures and Tables

**Table 1:** Binary logistic regression for main food shopping responsibilities in Spain.

	Odds Ratio	*p*	CI (95%)	
Gender *				
Males ^a^	0.221	< 0.001	0.178	0.276
Age ^†^ (years)				
18–30 ^b^	0.477	<0.001	0.327	0.697
31–49 ^b^	2.141	<0.001	1.514	3.026
50–64 ^b^	2.029	<0.001	1.393	2.955
65–75 ^b^	1.462	0.085	0.950	2.251

* Sample size: Males: 803; Females, 1223. ^†^ Sample size in age groups. >75 years, *n* = 227; 18–30 years, *n* = 320; 31–49 years, *n* = 801; 50–64 years, *n* = 460; 65–75 years, *n* = 218. a-Compared to female. b-Compared to >75 years.

**Table 2:** Binary logistic regression for spending 60 min or more on weekly food shopping in Spain.

	Odds Ratio	*p*	CI (95%)	
Gender *				
Males ^a^	0.755	0.014	0.605	0.944
Age ^†^ (years)				
18–30 ^b^	4.389	<0.001	2.579	7.471
31–49 ^b^	4.017	<0.001	2.548	6.335
50–64 ^b^	3.500	<0.001	2.175	5.632
65–75 ^b^	2.388	0.002	1.389	4.105

* Sample size: Males: 803; Females, 1223. ^†^ Sample size in age groups. >75 years, *n* = 227; 18–30 years, *n* = 320; 31–49 years, *n* = 801; 50–64 years, *n* = 460; 65–75 years, *n* = 218. a-Compared to female. b-Compared to >75 years.

**Table 3:** Preferences for food stores (%) in Spain.

	Fresh Products			Non-Perishable Products		
	Traditional market	Neighbourhood food stores	Supermarket supercentre	Traditional market	Neighbourhood food stores	Supermarket supercentre
Gender *						
Males	24.2 ^a^	26.0 ^a^	50.1 ^b^	3.3 ^a^	7.3 ^b^	89.5 ^c^
Females	27.0 ^a^	26.6 ^a^	46.7 ^b^	3.1 ^a^	6.8 ^b^	90.1 ^c^
Age ^†^ (years)						
18–30	19.2 ^a^	24.6 ^b^	56.2 ^c^	7.1 ^a^	5.8 ^a^	87.0 ^b^
31–49	22.6 ^a^	28.7 ^b^	48.7 ^c^	1.5 ^a^	4.6 ^b^	93.9 ^c^
50–64	29.6 ^a^	24.3 ^a^	46.0 ^b^	2.3 ^a^	6.9 ^b^	90.9 ^c^
65–75	31.4 ^a^	26.8 ^a^	41.8 ^b^	5.1 ^a^	13.4 ^b^	81.6 ^c^
>75	27.5 ^a^	23.5 ^a^	49.1 ^b^	6.1 ^a^	12.2 ^b^	81.8 ^c^

Different letter superscripts within the same row show statistical differences (*p* < 0.05). No differences were found for gender or between the different age groups compared to the reference age group (those >75 years). * Sample size: Males: 803; Females, 1223. ^†^ Sample size in age groups. >75 years, *n* = 227; 18–30 years, *n* = 320; 31–49 years, *n* = 801; 50–64 years, *n* = 460; 65–75 years, *n* = 218. a-Compared to female. b-Compared to >75 years.

**Table 4:** Reasons to choose the type of food store, for fresh and non-perishable food (%) in Spain.

	Fresh Products			Non-Perishable Foods		
Reasons to choose retail store	Traditional market *N* = 353	Neighbourhood food stores *N* = 370	Supermarket Supercentre *N* = 673	Traditional market *N* = 45	Neighbourhood food stores *N* = 99	Supermarket Supercentre *N* = 1270
Food quality	73 ^1,a^	68 ^1,a^	60 ^1,b^	49 ^1,a^	32 ^1,2,b^	25 ^1,b^
Proximity to home	22 ^2,a^	35 ^2,b^	45 ^2,c^	33 ^1,a^	43 ^1,a^	36 ^2,a^
Price	24 ^2,a^	18 ^4,b^	28 ^4,a^	35 ^1,a^	29 ^2,3,a^	34 ^2,a^
Convenience, home delivery	10 ^3,4,a^	10 ^5,a^	32 ^3,b^	29 ^1,2,a^	13 ^3,4,b^	34 ^2,a^
Freshness	21 ^2,a^	23 ^3,a^	13 ^6,b^	7 ^3,4,a^	7 ^4,5,a^	4 ^4,a^
Variety	14 ^3,a^	13 ^4,5,a^	21 ^5,b^	15 ^2,a,b^	7 ^4,5,b^	23 ^1,a^
Availability	7 ^4,a^	10 ^5,6,a^	12 ^6,a^	13 ^2,3,a^	6 ^4,5,a^	12 ^3,a^
Food origin	7 ^4,a^	7 ^6,a^	3 ^7,b^	7 ^3,4,a^	4 ^5,a,b^	2 ^5,b^
Reliance	7 ^4,a^	7 ^6,a^	1 ^8,b^	2 ^4,a,b^	5 ^5,a^	0.5 ^6,b^
Personalized service	3 ^5,a^	4 ^7,a^	0, 1 ^9,b^	0 ^4,a,b^	5 ^5,b^	0.3 ^6,a^
Routine habits	2 ^5,a^	1 ^8,a,b^	0, 6 ^8,9,b^	0 ^4,a,b^	2 ^5,a^	0.4 ^6,b^

Different letter superscripts show statistical differences (*p* < 0.05) within the same row; different number superscripts show statistical differences (*p* < 0.05) within the same column. *N* = 40 and *N* = 22 chose “other retail stores” for buying fresh foods and non-perishable food products respectively; *N* = 590 were non-respondents for this item.

**Table 5:** Binary logistic regression for habits/activities while shopping in Spain.

	OR	*p*	CI (95%)	
CHECKING THE “BEST BEFORE” DATE LABELS				
Gender *				
Males ^a^	0.666	0.008	0.492	0.901
Age ^†^ (years)				
18–30 ^b^	0.711	0.271	0.387	1.306
31–49 ^b^	1.073	0.787	0.643	1.793
50–64 ^b^	1.193	0.535	0.684	2.079
65–75 ^b^	1.372	0.360	0.697	2.701
PRICE COMPARISON				
Gender *				
Males ^a^	0.761	0.023	0.602	0.963
Age ^†^ (years)				
18–30 ^b^	3.173	<0.001	1.881	5.350
31–49 ^b^	1.846	0.001	1.275	2.673
50–64 ^b^	1.629	0.016	1.097	2.420
65–75 ^b^	1.845	0.012	1.146	2.973
READING NUTRITION FACTS LABELS				
Gender *				
Males ^a^	0.929	0.532	0.737	1.171
Age ^†^ (years)				
18–30 ^b^	2.368	0.001	1.454	3.854
31–49 ^b^	2.117	<0.001	1.466	3.058
50–64 ^b^	1.528	0.033	1.036	2.254
65–75 ^b^	1.172	0.491	0.746	1.841
LOOKING FOR FOOD DISCOUNTS AND COUPONS				
Gender *				
Males ^a^	0.616	<0.001	0.491	0.772
Age ^†^ (years)				
18–30 ^b^	3.953	<0.001	2.430	6.432
31–49 ^b^	3.075	<0.001	2.131	4.438
50–64 ^b^	2.569	<0.001	1.738	3.797
65–75 ^b^	1.769	0.013	1.126	2.781
LOOKING FOR HEALTHIER FOODS				
Gender *				
Males ^a^	0.767	0.104	0.557	1.056
Age ^†^ (years)				
18–30 ^b^	0.462	0.032	0.228	0.936
31–49 ^b^	0.604	0.108	0.327	1.117
50–64 ^b^	0.877	0.699	0.450	1.709
65–75 ^b^	0.855	0.690	0.396	1.847
BUYING ENVIRONMENTALLY FRIENDLY FOODS				
Gender *				
Males^a^	0.587	<0.001	0.472	0.730
Age ^†^ (years)				
18–30 ^b^	0.574	0.018	0.362	0.909
31–49 ^b^	0.611	0.009	0.422	0.883
50–64 ^b^	1.100	0.640	0.739	1.637
65–75 ^b^	1.616	0.053	0.995	2.624

* Sample size: Males: 803; Females, 1223. ^†^ Sample size in age groups. >75 years, *n* = 227; 18–30 years, *n* = 320; 31–49 years, *n* = 801; 50–64 years, *n* = 460; 65–75 years, *n* = 218. a-Compared to female. b-Compared to >75 years.

**Table 6:** Binary logistic regression for going fresh product shopping daily in the Spanish population.

	Odds Ratio	*p*	CI (95%)	
Gender *				
Males ^a^	0.93	0.646	0.683	1.267
Age ^†^ (years)				
18–30 ^b^	0.420	0.007	0.223	0.793
31–49 ^b^	0.452	0.001	0.287	0.712
50–64 ^b^	0.671	0.098	0.418	1.076
65–75 ^b^	0.686	0.192	0.390	1.208

* Sample size: Males: 803; Females, 1223. ^†^ Sample size in age groups. > 75 years, *n* = 227; 18–30 years, *n* = 320; 31–49 years, *n* = 801; 50–64 years, *n* = 460; 65–75 years, *n* = 218. a-Compared to female. b-Compared to > 75 years.

**Table 7:** Frequency of purchase of fresh products by habitat size (%) in Spain.

	Habitat Size (Inhabitants)					
Frequency	<2000 *N* = 173	2001 10,000 *N* = 347	10,001 100,000 *N* = 755	100,001 500,000 *N* = 482	>500,000 *N* = 269	*p*
Daily	7.7	11.6	15.7	16.7	13.4	0.060
Weekly or bi-weekly	92.3	88.4	84.3	83.3	86.6	0.081
*p*	<0.0001	<0.0001	<0.0001	<0.0001	<0.0001	<0.0001

**Table 8:** Fresh produce shopping by Nielsen Area (%) in Spain.

	Preferred Retail Food Stores			
Nielsen Zone	Traditional food market *N* = 353	Neighbourhood food stores *N* = 370	Supermarket/Supercentre *N* = 673	*p*
East Coast	26.9	25.5	47.6	0.013
South	30.9	25.4	43.7	0.005
Centre of Spain	16.0	39.6	44.3	0.005
Northwest	17.1	19.7	63.2	0.005
North	17.6	41.6	40.8	0.005
Barcelona (Metropolitan Area)	32.9	28.4	38.7	0.033
Madrid (Metropolitan Area)	30.7	20.6	48.7	0.005

**Table 9:** Binary logistic regression for the influence of Internet and traditional advertising on the food shopping habits in the Spanish population.

	Internet Advertising				Traditional Advertising			
	OR	*p*	CI (95%)		OR	*p*	CI (95%)	
Gender *								
Males ^a^	0.839	0.250	0.622	1.131	0.852	0.209	0.665	1.093
Age ^†^ (years)								
18–30 ^b^	9.200	<0.001	4.012	21.097	3.551	<0.001	2.010	6.276
31–49 ^b^	5.401	<0.001	2.475	11.786	3.327	<0.001	2.033	5.447
50–64 ^b^	2.937	0.010	1.296	6.659	1.637	0.068	0.964	2.783
65–75 ^b^	0.851	0.775	0.282	2.566	0.806	0.534	0.409	1.589

* Sample size: Males: 803; Females, 1223. ^†^ Sample size in age groups. >75 years, *n* = 227; 18–30 years, *n* = 320; 31–49 years, *n* = 801; 50–64 years, *n* = 460; 65–75 years, *n* = 218. a-Compared to female. b-Compared to >75 years.

## Supplementary Materials

The following are available online at www.mdpi.com/2072-6643/9/5/508/s1, Sociodemographic Data and Food Shopping Habits.
