# Supplementary material for: Present Food Shopping Habits in the Spanish Adult Population: A Cross-Sectional Study
Source: Nutrients. 2017 May 18;9(5):508. doi: 10.3390/nu9050508 (PMC5452238; doi:10.3390/nu9050508)
Supplement: Supplementary file 1 [file nutrients-09-00508-s001.zip › nutrients-188110-supplementary-final.pdf]

Present Food Shopping Habits in the Spanish Adult Population: A Cross-Sectional Study

María Achón [1], María Serrano [1], Ángela García-González [1], Elena Alonso-Aperte [1] and Gregorio Varela-Moreiras [1,2,*]

1. Questionnaire on Food Shopping Habits

1.1. *Sociodemographic Data*

**Q.1. Gender:**	code
Male	1
Female	2

**Q.2. How old are you? Age:**	code
18-30 y	1
31-49 y	2
50-64 y	3
65-75 y	4
>75 y	5

**Q.3. Where do you live?**<br>**Province:** ________________<br>**City/Village:** _____________<br>Habitat size	code
< 2,000 inhabitants	1
2,001-10,000 inhabitants	2
10,001-10,000 inhabitants	3
100,001-500,000 inhabitants	4
> 500,000 inhabitants	5

**Q.4. Nationality**	code
Spanish	1
Other: ___________	2

**Q.5. Employment Status: Are you currently…?**	code
Self-employed	1
Working for others	2
Housewife (domestic work)	3
Unemployed (Go to Question Q.5.1)	4
Student	5
Retired	6
Other: __________________________	7

**Q.5.1. For how long you have been unemployed?**	
______________________	

1.2. *Food Shopping Habits*

**Q.6. Is there a food store in your living area?**	code
Yes	1
No	0

**Q.7. Are you the person in charge of food shopping at your home?**	
I am 100% in charge	1
I am 50% in charge	2
I am < 50% in charge	3
I am in charge occasionally	4
I am not involved in food shopping	5

If Q.7 = 1 or 2, go to Q.8
If Q.7 ≠ 1 or 2, you are finished.

**Q.8. Which is your monthly budget for food shopping?**	
<150 euros	1
150-300 euros	2
301-450 euros	3
451-600 euros	4
> 600 euros	5

**Q.9. Have you recently changed your shopping habits due to the current economic situation?**	YES	NO
I buy cheaper food products	1	0
I buy more generic or distribution brand	1	0
I look for low-cost food- shops	1	0

I cook at home and take my lunch-box to work	1	0
I do not eat out so often	1	0
I skip one meal (__________________)	1	0
I buy less fresh foods (fruits, vegetables)	1	0
I buy less meat and fish	1	0
I don't go out for snaking or appetizers	1	0
I don't go out to have a drink	1	0
I had to ask for food aid support	1	0

**Q.10.Do you buy different foods depending on the day of the month (beginning vs end)?**	code
Yes	1
No	0

**Q.11. Do you buy in different shops depending on the day of the month (beginning vs end)?**	code
Yes	1
No	0

**Q.12. How frequently do you buy fresh foods? (one choice)**	code
Daily	1
3-4 times per week	2
2-3 times per week	3
Once a week (Monday to Friday)	4
Usually on weekends	5
Biweekly	6
Once a month	7
Sporadically	8

**Q.13. How frequently do you buy non-perishable foods? (one choice)**	code
Daily	1
3-4 times per week	2
2-3 times per week	3
Once a week (Monday to Friday)	4
Usually on weekends	5
Biweekly	6
Once a month	7
Sporadically	8

**Q.14. Where do you prefer to buy fresh foods? (one choice)**	code
Traditional Markets	1
Neighbourhood food stores	2
Supermarkets	3
Supercentres	4
24h Shops	5
Internet	6
Direct from suppliers	7
Organic food shops	8
Own production	9
Other (__________________________)	10

**Q.15. Where do you prefer to buy non-perishable foods? (one choice)**	code
Traditional Markets	1
Neighbourhood food stores	2
Supermarkets	3
Supercentres	4
24h Shops	5
Internet	6
Direct from suppliers	7
Organic food shops	8
Own production	9
Other (__________________________)	10

**Q.16. What are the reasons why you prefer that kind of retail store to buy your fresh food items? (multiple choice).**	code
Because of food product quality	1
Because of variety	2
Because of the price	3
Because of freshness	4
Because of availability	5
Because of proximity to home	6
Because of convenience (home delivery)	7
Due to the products´ origin	8
Other (__________________________)	9

**Q.17. What are the reasons why you prefer that kind of retail store to buy your non-perishable food items? (multiple choice).**	code
Because of food product quality	1
Because of variety	2
Because of the price	3
Because of freshness	4
Because of availability	5
Because of proximity to home	6
Because of convenience (home delivery)	7
Due to the products´ origin	8
Other (__________________________)	9

**Q.18 Which of the following habits/activities do you usually perform while food shopping??**	YES	NO
You check the "best before" date label	1	0
You check the nutritional facts label	1	0
You bring a food shopping list with you	1	0
You choose environmentally friendly foods	1	0
You look for food discounts and coupons	1	0
You compare prices	1	0
You look for healthier foods	1	0
You buy value-added- foods (omega-3 enriched; + bifidus)	1	0

**Q.19. How much time do you spend on food shopping (from "leaving home" to "food in the fridge") (min)**
____

**Q.20. Do you feel you have enough time for food shopping and cooking meals?**	code
Yes	1
No	0

**Q.21. Does traditional food advertising (TV, radio, press) have an influence on your shopping habits?**	code
Yes	1
No	0

**Q.22. And the information on the Internet and Social Media, does it influence your shopping habits?**	code
Yes	1
No	0

**Q.23. Please rate from 1 to 10 how health consciousness influences your food shopping habit, being 1 "no influence at all" and 10 "very high influence".**									
No influence at all		Very small influence		Intermediate		High influence		Very high influence	
1	2	3	4	5	6	7	8	9	10

© 2016 by the authors; licensee MDPI, Basel, Switzerland. This article is an open access article distributed under the terms and conditions of the Creative Commons by Attribution (CC-BY) license (http://creativecommons.org/licenses/by/4.0/).
